# Infertility, the outcomes of assisted reproductive technologies use in childhood cancer survivors: a scoping review

**DOI:** 10.1007/s11764-026-01988-5

**Published:** 2026-02-16

**Authors:** Elizabeth Sorial, Leah Roberts, Erin Kelty, Sarah V. Ward, Ying Ru Feng, David B. Preen

**Affiliations:** 1 School of Population and Global Health, The University of Western Australia, 35 Stirling Hwy, Crawley, WA 6009 Australia; 2 UWA Medical School, The University of Western Australia, 35 Stirling Hwy, Crawley, WA 6009 Australia

**Keywords:** Childhood cancer survivors, Paediatric cancer, Assisted reproductive technology, Infertility, Perinatal outcomes, Fertility preservation

## Abstract

**Purpose:**

Childhood cancer survivors (CCS) are at increased risk of treatment-related infertility and may require assisted reproductive technologies (ART) to achieve parenthood. This scoping review aimed to synthesise the existing scientific evidence on the use of ART among CCS, focusing on fertility outcomes and associated maternal, perinatal, and child health indicators.

**Methods:**

Adapted from Joanna Briggs Institute (JBI) and PRISMA-ScR guidelines, MEDLINE (OVID), CINAHL, and Embase were searched (conducted from the inception of each database up to February 2025) for studies of CCS diagnosed before age 18, surviving ≥ 5 years, and reporting ART-related fertility, obstetric, or perinatal outcomes. Screening and extraction were conducted independently via Covidence.

**Results:**

From 4,785 records, 10 studies met the inclusion criteria. Five studies reported higher infertility rates among female CCS (4.5–34%) compared with non-cancer controls. ART success rates were generally comparable to siblings, though pelvic or cranial radiotherapy reduced live birth rates by 17–32%. Three studies demonstrated the feasibility of ovarian tissue cryopreservation (OTC), particularly for prepubertal girls. Male CCS were more likely to require intracytoplasmic sperm injection (ICSI) and had lower ART success than non-cancer siblings. Neonatal studies found increased multiple gestations, preterm births, and low birth weight among ART-conceived offspring, but no rise in congenital anomalies or childhood cancer.

**Conclusion:**

ART enables parenthood for childhood cancer survivors, though prior gonadotoxic therapies can impair success and increase perinatal risks, highlighting the need for long-term survivorship research.

**Implications for Cancer Survivors:**

ART provides viable fertility options, but early counseling and integrated reproductive care are essential to optimise outcomes and ensure equitable access for all cancer survivors.

**Supplementary Information:**

The online version contains supplementary material available at https://doi.org/10.1007/s11764-026-01988-5.

## Introduction

Over the past five decades, substantial progress in the diagnosis and treatment of paediatric malignancies has led to marked improvements in survival outcomes. In high-income countries such as the United States, Canada, and across Europe, the 5-year survival rate for childhood cancer now exceeds 80% [1, 2]. As a result of these advancements, the global population of childhood cancer survivors (CCS) has expanded considerably, with recent estimates indicating that over 500,000 individuals have survived childhood cancer worldwide [3, 4]. This growing cohort has prompted increasing attention on the long-term health consequences following cancer and its treatment during childhood and adolescence, highlighting the need for comprehensive survivorship research to inform lifelong clinical care and public health planning [1].

Many CCS require ongoing medical services throughout their life, due to the late effects of cancer and/or its treatment. Medical complications following childhood and adolescent cancers are more often attributable to the long-term toxicities of radiation and chemotherapy, rather than surgical interventions, with late effects emerging months to decades after treatment [5]. These late effects can include impaired growth and development, dysfunction of bodily organs or systems, increased risk of subsequent cancers, and fertility problems [5].

The reproductive system is particularly vulnerable to the negative impacts of cancer therapies. For example, alkylating agents and radiation can damage ovarian and testicular tissue, leading to reduced fertility or infertility [6]. In fitting with this, studies have documented higher rates of infertility, premature ovarian failure, and early menopause among female CCS compared to non-cancer controls [7, 8]. As such, fertility preservation and reproductive health support become critical aspects of care for CCS [9].

Fertility challenges can be a significant source of distress, potentially impacting survivors’ quality of life and mental health, and have been associated with depression, anxiety, and trauma [6]. Despite the importance of fertility to many CCS and their families, research on survivors’ perceived reproductive health needs and long-term outcomes remains limited [10]. Further, CCS are increasingly utilising ART to achieve parenthood [11]. However, the use of ART has been associated with increased risk of adverse maternal and perinatal outcomes in the general population [12–14]. Given the complex health profiles of CCS, the potential for increased risks warrants further investigation.

Several studies have reported on CCS expressing concern regarding the potential health implications of their prior cancer treatment on future offspring, including fears of increased risk of cancer and other adverse health outcomes [12, 15]. Existing studies have reported that children born to survivors of non-hereditary childhood cancer may be at elevated risk of congenital malformations, genetic disorders, or non-hereditary malignancies [14]. As an increasing number of CCS pursue parenthood via ART, it is critical to understand whether ART may further compound these risks. Despite this emerging clinical relevance, there have been few studies investigating ART-related health outcomes in this population and, to date, the literature in this area has not been reviewed [16, 17]. Therefore, this scoping review aimed to identify and synthesise the existing evidence on obstetric, birth, and child health outcomes associated with fertility preservation and other ART-related techniques among CCS.

## Methods

**Fig. 1:**
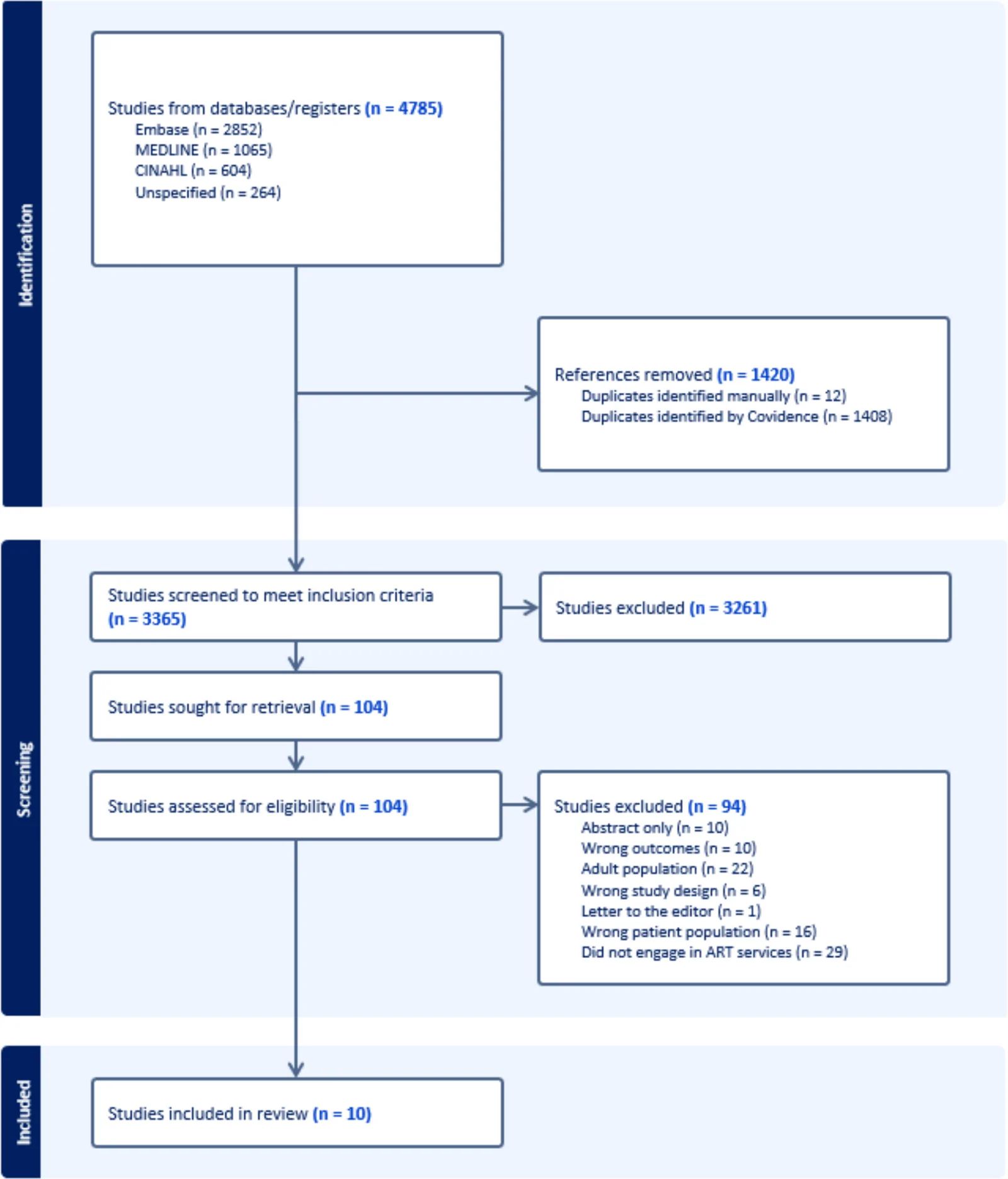
Analyses (PRISMA) flow chart for the selection of studies included in the review

The review was reported according to the Preferred Reporting Items for Systematic Reviews and Meta-Analyses extension for Scoping Reviews (PRISMA-ScR) checklist and prospectively registered at Open Science Framework on 8 April 2024. The study selection process is summarised in Fig. 1.

### Search strategy

Search terms were developed to capture concepts related to CCS and ART. Both Medical Subject Headings (MeSH) and free-text terms were used, tailored to each database. The following main concepts guided the search: *Population (childhood/early-onset/adolescent cancer survivors), Exposure/Intervention (fertility, ART, fertility preservation techniques), Outcomes (reproductive, perinatal, maternal, neonatal, congenital outcomes), and Database-specific indexing (MeSH for cancer, paediatrics, female, fertility, ART, chemotherapy, radiotherapy, cryopreservation).* A full list of search terms and search strategies for each database is available in Supplementary Table 1. We identified studies by searching MEDLINE (OVID), Embase, and CINHAL, encompassing literature from peer-reviewed journal articles.

### Eligibility criteria

Studies were eligible for inclusion if the study sample included paediatric/childhood cancer survivors diagnosed before the age of 18 years, reported on the utilisation of fertility services, assessed perinatal and/or obstetric outcomes and were written in English. Excluded studies included case-reports, animal studies, conference abstracts or studies where no statistical comparisons were made. Eligible study designs included case collections of CCS only, as well as case–control studies. No restrictions were applied regarding the type of control group; comparators included siblings of CCS, individuals with non-ART conceptions, and CCS who did not undergo ART The date range of studies published between 1974–2025 were included in the search.

### Screening process

Databases were searched using Ovid Technologies, with identified publications downloaded into Endnote (*n* = 4785) (Fig. 1). Endnote functions were used to remove duplicate records, with the remaining studies uploaded to Covidence systematic review software [18] where any additional duplicate records were removed (*n* = 1420). Two trained reviewers (ES and LR) independently screened titles and abstracts of identified studies to determine if the studies met the inclusion criteria (*n* = 3365). Subsequently, full-text screening of likely eligible studies (*n* = 104) was independently undertaken by three reviewers (ES, ER, LR), and conflicts were resolved via discussion.

### Data extraction

Data charting was conducted independently in Covidence and extracted to Microsoft Excel for the final 10 studies included in the review (Fig. 1). Information collected included the ART service used, cancer type, cancer treatment type, outcome measures (such as perinatal, birth, and adverse obstetric), comparison group, article characteristics (e.g. first author and year of publication), and study design.

## Results

### Maternal fertility preservation and ovarian tissue cryopreservation

The only fertility preservation method currently available for pre-pubertal girls is ovarian tissue cryopreservation (OTC). OTC is also an important option for adolescent and adult females who are unable to undergo oocyte or embryo cryopreservation [19, 20]. There were three studies that examined OTC in CCS [21–23]. Across these studies, a total of 2,698 patients were counselled for and/or underwent OTC, with consistent evidence supporting the safety and effectiveness of this method across age groups and indications. However, in two of these three studies, paediatric and adolescent patients (< 18 years) made up only a small proportion of patients included in the study (14 and 28%) [21, 23]. In contrast, all patients included in the study by Poirot et al. were under 15 years of age (*n* = 418). Common malignancies included lymphoma, leukemia, sarcomas, and neuroblastoma [21–23] 

Fabbri et al. examined OTC outcomes in CCS, identifying significantly higher follicular density in paediatric patients compared with adults (127.2 vs. 19.1 follicles/mm^2^, *p* < 0.01) [21]. Histological analyses in cohorts with mean ages of 14.2 and 30.0 years confirmed strong structural preservation of follicles in both fresh and thawed ovarian tissue samples [21]. Although the proportion of stored tissue that was ultimately utilised was low, the authors demonstrated clinical relevance through reported transplantations and subsequent reproductive outcomes [21]. Specifically, 33 ovarian tissue transplantations were performed in 24 women, representing a transplantation proportion of 2%, with a mean storage duration of 7.84 ± 3.5 years (range 2–17 years) [21]. Among women with premature ovarian insufficiency, restoration of ovarian function was achieved in 100% of those undergoing heterotopic transplantation (*n* = 4) and 85% of those receiving orthotopic transplantation (*n* = 11) [21]. Six pregnancies were reported, resulting in four live births and two miscarriages, corresponding to a live birth proportion of 67% [21].

In contrast, Rodriguez-Wallberg et al. reported broader utilisation of fertility preservation services and subsequent pregnancy attempts, rather than OTC-specific outcomes [23]. In this cohort, 37 of 75 women (49%) returned to attempt pregnancy after a mean interval of 3.9 years following fertility preservation. Live births were achieved in 21% of women with a prior malignancy compared with 47% of those with benign indications (*p* = 0.002) [23]. These findings reflect outcomes among women with a history of cancer more generally and are not limited to childhood cancer survivors [23].

### Fertility and reproductive outcomes post cancer

Studies into fertility and reproductive outcomes in CCS were rare, with two studies identified [24]. Barton et al. identified female CCS faced significantly higher infertility risks than their siblings, with a 48% increased risk of clinical infertility (RR 1.48, 95% CI 1.23–1.78) [24]. The greatest disparity observed was among those aged ≤ 24 years, who experienced nearly a threefold higher risk of infertility than their siblings who had not experienced cancer (RR 2.92, 95% CI 1.18–7.20) [24]. When treatment exposures were examined, infertility risk was elevated for both uterine radiation and alkylating chemotherapy [24]. For uterine radiation, the association was significant across all dose categories but appeared to decrease slightly with higher doses: RR 2.48 (95% CI 1.54–4.01) for 5.1–10.0 Gy, RR 2.02 (95% CI 1.27–3.23) for 10.1–20.0 Gy, and RR 1.95 (95% CI 1.19–3.19) for > 20.0 Gy [24]. This pattern may reflect small numbers in higher-dose groups, survivor selection bias, or misclassification of radiation fields, rather than a true inverse dose–response relationship [24]. Exposure to alkylating chemotherapy was also associated with infertility (RR 1.48, 95% CI 1.10–1.99) [24].

Borgmann-Staudt et al. showed among 1,659 offspring of childhood cancer survivors (CCS), 74 (4.5%) were conceived via assisted reproductive technologies (ART) compared with 3.7% in siblings, indicating slightly greater ART use among survivors [17]. Infertility was reported in 40% of CCS couples, most often due to male factors (59%), consistent with treatment-related gonadotoxicity [17]. ICSI was the predominant technique (56%), with most using fresh gametes (77%) [17]. Over half (52%) conceived after one ART cycle, and 9% required ≥ 4 cycles, reflecting generally favourable fertility outcomes post-cancer [17]. Pregnancy complications occurred in 31% of ART pregnancies, but perinatal outcomes were reassuring: preterm birth (21.9%) and low birth weight (17.6%) were linked to multiple births rather than ART itself [17]. No increased risk of congenital anomalies, heart defects, or childhood cancer was observed, suggesting comparable reproductive success and offspring health in CCS using ART [17].

Melin et al. assessed differences in live birth rates (LBR) following fresh in vitro fertilisation (IVF)/intracytoplasmic sperm injection (ICSI) cycles using autologous oocytes between female survivors of early-onset cancer and their cancer-free sisters [25]. Among the 8944 female cancer survivors included in the cohort, 1128 (12.6%) were diagnosed with cancer during childhood (0–14 years) [25]. Of these, 19 childhood cancer survivors underwent fresh IVF/ICSI treatment between 1993 and 2012 [25]. Within this subgroup, five individuals (26.3%) achieved a live birth within 22–48 weeks following a fresh IVF/ICSI cycle [25]. When compared to sibling controls, there was no difference in LBR between the two groups (adjusted RR 0.78, 95% CI 0.33–1.84) [25]. Due to the small number of events, estimates were imprecise. Nonetheless, the findings suggest that childhood cancer survivors who proceed to IVF/ICSI may have comparable probabilities of live birth to their non-cancer siblings.

### ART outcomes in female CCS

Keefe et al. reported that among CCS who used autologous oocytes without gestational carriers, 97 individuals underwent 155 stimulations, resulting in 49 live births [26]. This corresponded to a LBR of 31.6% per ovarian stimulation cycle, which was not significantly different from cancer-free siblings (38.3%; *p* = 0.39) [26]. Similarly, the LBR per embryo transfer was 43.9% in survivors versus 50.0% in female siblings (p = 0.33) [26]. Exposure to cranial (RR, 0.44; 95% CI, 0.20–0.97) or pelvic radiation therapy (RR, 0.33; 95% CI, 0.15–0.73) was associated with a significantly reduced likelihood of live birth compared to siblings [26]. In contrast, high-dose alkylator exposure (cyclophosphamide-equivalent dose ≥ 8000 mg/m^2^) was not significantly associated with live birth likelihood (RR, 1.04; 95% CI, 0.52–2.05) [26].

### Paternity and ART use in male survivors

Studies by Claessens et al. and Kitlinski et al. consistently reported that male CCS have lower rates of natural conception and higher reliance on ART compared to their siblings without cancer [27, 28]. Claessens et al. found that 65% of male CCS fathered a biological child, with survivors being three times more likely to use ART than their same-sex siblings [27]. Similarly, Kitlinski et al. reported that CCS were more likely to achieve paternity through ICSI (adjusted OR (aOR) 3.52, 95% CI 2.52–4.93) but not through conventional IVF (aOR 1.02, 95% CI 0.61–1.70), compared with natural conception [28]. Despite their increased use of ART, CCS still had significantly lower birth rates compared to non-cancer siblings after ART (*p* = 0.001) [28].

Claessens et al. reported that fewer male CCS fathered biological children compared to their siblings without cancer (56% vs. 88%; *p* < 0.001) [27]. The method of conception among those who did father a child was comparable between CCS and siblings, with spontaneous conception occurring in 90% of both groups [27]. However, CCS were more likely to seek reproductive assistance due to difficulties conceiving (34% vs. 12%, respectively; *p* < 0.001) [27]. Despite this finding, fewer CCS who sought fertility treatment proceeded with ART compared to siblings (41% vs. 77%, respectively; *p* = 0.001), and those who did had lower success fractions in achieving paternity (49% vs. 94%, respectively) [27]. Kitlinksi et al. found that only survivors of haematological and lymphatic cancers had significantly higher odds of fatherhood through IVF compared with fathers without a cancer diagnosis (aOR 1.98, 95% CI 1.10–3.56) [28].

### Perinatal outcomes following ART in CCS

Two cohort studies were identified that examined perinatal outcomes following ART in female CCS [16, 17]. In both cohorts, children born to survivors using ART exhibited increased rates of adverse perinatal outcomes compared to spontaneously conceived offspring of survivors [16, 17]. Borgmann-Staudt et al. observed that ART-conceived neonates of CCS females were significantly more likely to be born preterm (< 37 weeks gestation; 21.9% vs. 10.1%, OR = 2.50, 95% CI: 1.40–4.46) and to have a low birth weight (< 2500 g; 17.6% vs. 6.6%, OR = 3.04, 95% CI: 1.62–5.71) compared with naturally conceived pregnancies in CCS [17]. Multiple births were also substantially higher in ART-conceived children compared with spontaneously conceived children of survivors (29.7% vs. 2.5%, *p* < 0.001) [17]. However, when adjusting for covariates such as multiple births, maternal smoking, and parental cancer diagnosis, ART was not seen to be independently associated with low-birth weight or preterm birth [17]. Sommerhauser et al. reported significantly elevated levels of multiple births (28.1% vs. 3.0%, *p* < 0.001) and low birth weight (15.8% vs. 6.6%, OR = 2.66, 95% CI: 1.26–5.62) among ART-conceived offspring compared to spontaneously conceived offspring [16]. Mean gestational age was slightly lower in the ART group (38.5 vs. 39.1 weeks, *p* = 0.028) compared with spontaneously conceived children of survivors, but the prevalence of preterm birth did not differ significantly [16]. However, the association between ART and low birth weight of children to CCS females did not remain significant after adjusting for known confounders including multiple births and preterm delivery [16].

### Maternal obstetric outcomes

Sommerhauser et al. reported pregnancy complications and modes of delivery among childhood cancer survivors who conceived using ART. Among female survivors, 31% experienced complications such as gestational diabetes or premature contractions, and most (65%) had spontaneous vaginal deliveries [16]. However, these obstetric outcomes were only described within the ART group and were not compared against survivors who conceived spontaneously or against population controls, limiting interpretation of relative risk [16] Table 1.

**Table 1:** Summary table of clinical literature meeting the review inclusion criteria

First author, year	Study location	Study design	Study aims	Control group (*n*)	Number of childhood cancer survivors (*n*)	Summary of relevant findings
Barton et al. 2013	United States of America	Cohort study	Investigated infertility and time to pregnancy in female childhood cancer survivors, and analysed treatment characteristics associated with infertility and subsequent pregnancy	Sibling control group *n* = 1366	*n* = 3531	• ↑ Higher risk of clinical infertility among survivors, especially at younger reproductive ages • Similar rates of seeking infertility treatment compared to controls • ↓ Lower likelihood of receiving fertility drugs • Infertility strongly associated with prior uterine radiation and alkylating chemotherapy • ↑ Longer time to pregnancy in survivors
Borgmann-Staudt et al. 2022	Germany, Austria, Czech Republic, Poland and Switzerland	Explorative, retrospective cohort study,	The study compared: (i) the prevalence of ART utilisation in survivors and their siblings, (ii) perinatal outcomes, malformations, heart defects and cancer in ART-conceived survivor offspring with those conceived spontaneously and (iii) the outcomes of survivor offspring vs. sibling offspring	Sibling offspring cohort *n* = 387	*n* = 1681	• ↑ Slightly higher ART use among survivors than siblings • Most survivors achieved pregnancy after one ART cycle, typically via ICSI using fresh oocytes and sperm • ↑ Higher rates of multiple births in ART-conceived pregnancies among survivors • ↑ Increased risk of low birth weight in ART pregnancies (unadjusted analyses) • ↑ Higher likelihood of preterm birth in ART pregnancies (unadjusted analyses) • No increased risk of childhood cancer, congenital anomalies, or heart defects associated with ART
Claessens et al. 2024	The Netherlands	Cohort Study	Evaluated reproductive outcomes and reproductive health care utilization among male CCS compared with male siblings	Male siblings *n* = 185	*n* = 1317	• ↓ Fewer CCS had biological children compared to their siblings • Spontaneous conception rates among fathers were similar • More CCS sought reproductive specialist consultations • ↓ Fewer CCS proceeded with ART after consultation • ↓ Lower likelihood of fathering a child via ART among CCS
Fabbri et al. 2022	Italy	Retrospective cohort study	Reported outcomes from ovarian tissue cryopreservation (OTC) and ovarian tissue transplantation (OTT) in paediatric and adult patients, both with and without cancer diagnoses, at a clinical centre, over a 20-year time-period	*n* = 96	*n* = 930	• ↑ Higher follicular density in paediatric patients compared to adults • Good preservation of follicles and ovarian tissue observed across all age groups in both fresh and thawed samples • Ovarian tissue cryopreservation enabled fertility restoration • Six pregnancies achieved, resulting in four healthy births
Keefe et al. 2020	US	Retrospective cohort study	Investigated ART cycle characteristics, including live birth outcomes of childhood cancer survivors who underwent ART compared with siblings who also underwent ART	Sibling control group *n* = 2419	*n* = 9885	• Live birth rate per stimulation was slightly lower in survivors using autologous eggs without gestational carriers • Live birth rate per embryo transfers also lower in survivors • ↓ Prior cranial and pelvic radiation were associated with reduced live birth rates • Alkylating agent exposure had no significant effect on live birth outcomes
Kitlinski et al. 2023	Sweden	Cohort study	This study examined how a history of cancer influenced the likelihood of using assisted reproductive technologies (ART) to achieve paternity	Fathers with no cancer diagnosis *n* = 1,154,587	*n* = 26,901	• ↑ Men with a cancer history, especially testicular, prostate, or haematological cancers, were more likely to use assisted reproduction to achieve paternity compared to men without cancer history
Melin et al. 2021	Finland	Retrospective, register-based study	This study assessed whether the probability of a live birth following fresh IVF/ICSI cycles with autologous oocytes differed between women with a history of early-onset cancer and their siblings	*n* = 9848	*n* = 8944	• Female cancer survivors had similar live birth rates as siblings after four fresh IVF/ICSI cycles, including early-onset cases • ↓ Overall live birth rate was lower in survivors compared to siblings
Poirot et al. 2019	France	Retrospective study	This study described 20 years of experience with fertility preservation through ovarian tissue cryopreservation (OTC) in children up to 15 years of age, including the collection of isolated oocytes during the procedure, patient characteristics, and cohort follow-up	*n* = 105	*n* = 313	• Three patients used their ovarian tissue, one for puberty induction, two for fertility restoration • No pregnancies have been achieved from the transplanted tissue
Rodriguez-Wallberg et al. 2019	Sweden	Prospective study	This study investigated trends in female patients’ choices following counselling, as well as fertility preservation outcomes during follow-up, in relation to benign versus malignant indications	N/A	*n* = 178	• ↓ OTC has lower utilisation rates compared to embryos and oocytes overall • Increasing number of teenagers now opt for oocyte cryopreservation via controlled ovarian stimulation, either before or after cancer treatment
Sommerhauser et al. 2021	Germany	Cohort study	This study compared perinatal outcomes and the prevalence of childhood cancer and congenital malformations, including heart defects, in children born to German cancer survivors following ART and after spontaneous conception	*n* = 2478	*n* = 1340	• ART was successful in childhood cancer survivors using both fresh and cryopreserved oocytes/sperm • No adverse impact on perinatal or health outcomes when adjusting for known confounders

## Discussion

This review synthesised available evidence on reproductive outcomes following cancer treatment in CCS, with the aim of clarifying the impact of cancer and its treatment on fertility and broader reproductive health. It identifies key evidence gaps beyond conception and highlights the limited research currently available in this area, in particular maternal obstetric outcomes. Clinical infertility was more prevalent among CCS, particularly at younger reproductive ages, and strongly associated with prior exposure to alkylating agents and uterine radiation [17]. Despite similar rates of fertility consultation, survivors were less likely to receive pharmacologic treatment, suggesting underlying barriers to care, such as cost, perceived success of treatment, age, and knowledge [29]. Nonetheless, many ultimately achieved pregnancy, often via ART, with ICSI being the most used modality [25]. While ART use was only modestly elevated in this group, survivors demonstrated variable success, with male survivors in particular experiencing poorer outcomes [26–28]. ART-conceived pregnancies in CCS were associated with increased risks of multiple births compared to spontaneous pregnancies in CCS [14, 17, 25]. Inconsistent evidence was observed for increased incidence of preterm delivery and low birthweight amongst ART-conceived pregnancies in CCS and no elevated risks of congenital anomalies or childhood cancers were observed. Fertility preservation through OTC appears promising for prepubertal patients, but its utilisation remains limited and long-term efficacy is not well established [30]. These findings underscore the need for comprehensive, longitudinal research encompassing not only fertility but also obstetric, perinatal, and child health outcomes, and highlight the importance of early, individualised fertility counselling within survivorship care.

### Maternal fertility preservation and ovarian tissue cryopreservation

There is consistent, though limited, evidence that OTC remains the only feasible fertility preservation option for pre-pubertal girls with cancer [21–23]. While OTC preserves ovarian function and can enable puberty induction and potential fertility restoration, data on live births remain limited and overall utilisation rates are low, particularly when compared with post-pubertal patients for whom oocyte or embryo cryopreservation is the preferred strategy [21–23]. Although higher follicular density has been observed in younger patients, its translation into successful pregnancies remains uncertain [21]. Persistent disparities in access between high- and low-income settings further underscore the need for standardised protocols and longitudinal, CCS-specific research to clarify reproductive and psychosocial outcomes following OTC [21, 31].

The findings from Fabbri et al. underscore the theoretical advantage of earlier tissue harvesting, suggesting enhanced preservation potential at younger ages [21]. However, the translation of this biological potential into successful pregnancies remains uncertain due to limited long-term data. Additionally, other studies have suggested that disparities in OTC access and utilisation, particularly between high-income and low-to-middle-income countries, result in systemic inequities in fertility preservation care [21, 31]. This highlights the need for standardised guidelines, improved accessibility, and longitudinal studies to assess reproductive and psychosocial outcomes following OTC for CCS.

### Fertility and reproductive outcomes post-cancer

Female CCS experience significantly higher rates of clinical infertility compared to their peers without a childhood cancer diagnosis; with the disparity most pronounced at 0–4 and 5–9 years of age at diagnosis [24]. This pattern highlights the early and potentially enduring impact of cancer therapies on reproductive potential [24]. Despite these challenges, the findings also reveal that many survivors ultimately achieve pregnancy, suggesting that infertility in this group may be overcome with time or intervention [24]. However, delays in time to pregnancy and reduced access to fertility treatments further compound reproductive difficulties for this population, underscoring the need for early identification of at-risk individuals and timely referral to reproductive care [24]. While survivors were equally likely to seek medical evaluation for infertility, they were substantially less likely to receive pharmacologic treatment, suggesting the presence of barriers to care, such as cost, age, knowledge, and access [32]. Despite this finding, a substantial proportion of survivors with self-reported clinical infertility (64%) ultimately achieved pregnancy, highlighting the potential for successful conception even among high-risk individuals and the value of sustained reproductive counselling and support for CCS [24]. However, it is important to note that these findings are derived from a single study, which may limit the generalisability of conclusions. This underscores the need for further research to confirm and extend these observations across diverse populations and healthcare settings.

Importantly, infertility was strongly associated with specific treatment exposures, including uterine radiation and high cumulative doses of alkylating agents, reinforcing the critical need for early, individualised fertility preservation counselling at the time of cancer diagnosis. These findings highlight that the presence of menstruation alone is an inadequate indicator of fertility potential, underscoring the importance of risk stratification based on prior treatment exposures. Furthermore, the observed disparity in the prescription of fertility medications warrants further investigation to ensure equitable access to fertility care among CCS.

### ART outcomes in female CCS

Although ART success rates in female CCS appear broadly comparable to those of the general population, prior exposure to pelvic or cranial radiotherapy was reported to be associated with reduced LBR in this population [25]. Keefe et al. observed significantly reduced live birth rates among survivors who had received these modalities, suggesting a need for specialised ART protocols in this subgroup. Despite this finding, survivors previously treated with alkylating agents should not be excluded from ART consideration, as findings indicated that they could still achieve live births using autologous oocytes [26].

While an overall reduction in LBR was observed among survivors, this was independent of IVF/ICSI treatment, suggesting a general decline in reproductive potential in this group [25]. Notably, among those who were offered and proceeded with IVF/ICSI, the probability of a live birth was comparable to that of non-cancer siblings. These findings may reflect a selection bias, where CCS undergoing ART represent a subgroup with more favourable reproductive potential. This may include those with reassuring clinical indicators (i.e. regular menstruation, normal anti-mullerian hormone levels who are more likely to be referred or choose to pursue ART. As such, ART use in CCS may not reflect the broader survivor population but rather those perceived by themselves or clinicians to have greater chances of success, potentially skewing outcomes. The study highlights the importance of personalised oncofertility counselling as part of routine care for young females with cancer, not only to discuss fertility preservation prior to gonadotoxic treatment but also to monitor reproductive function over time and provide timely fertility treatment when indicated.

### Paternity and ART use in male survivors

Evidence from two studies on paternity outcomes in male CCS highlights the long-term reproductive consequences of cancer and its treatment, reinforcing concerns about impaired fertility in this population [27, 28]. Both studies demonstrated that male CCS have lower rates of spontaneous conception and are more likely to require ART to achieve fatherhood, compared with fathers with no prior cancer diagnosis [27, 28]. The success of ICSI among survivors with malignancies involving the male reproductive tract or haematological and lymphatic systems suggests that ART can be effective, but outcomes are correlated with prior cancer therapy type [24, 28]. Importantly, even among those male CCS seeking fertility treatment, survivors were less likely to proceed with, or have a successful outcome from, ART when compared to their same-sex siblings without cancer.

These findings support the need for early, tailored fertility counselling, particularly for male CCS at high risk of infertility. Additionally, the lower ART uptake among CCS, despite higher consultation rates, raises concerns about access, affordability, or provider-related barriers to care that warrant further investigation.

### Perinatal outcomes following ART in female CCS

Limited evidence suggests that female childhood cancer survivors who conceive using ART may have higher rates of preterm birth and low birth weight compared to survivors who conceive spontaneously [16, 17]. However, these associations appear largely attributable to higher rates of multiple births and maternal factors, as adjustment for these covariates attenuated the observed risks. Similar patterns have been reported in the general ART population, suggesting that ART in CCS does not independently increase perinatal risk beyond that expected for ART users generally. Future research should account for plurality, maternal characteristics, and treatment exposures to better clarify perinatal risk profiles in this population.

Other than preterm delivery and low birth weight, evidence on perinatal and neonatal risks associated with ART in CCS remains limited. While ART in the general population has been linked to higher rates of NICU admissions and lower Apgar scores [33], comparable data for children of CCS are lacking. Existing studies have focused mainly on immediate perinatal outcomes among ART-conceived offspring of CCS but have not compared these with outcomes in non-CCS who conceived through ART. This gap limits understanding of whether CCS experience similar or distinct ART-related complications. Beyond the perinatal period, few studies have examined longer-term infant or child health outcomes in this group, despite evidence from the general population suggesting potential risks associated with ART, including neurodevelopmental and metabolic conditions [13]. Future studies directly comparing ART outcomes between CCS and non-CCS populations are needed to clarify whether a history of cancer modifies short- or long-term risks associated with ART [13].

### Maternal obstetric outcomes

No studies were identified with a primary focus on assessing maternal obstetric outcomes among female CCS undergoing ART, such as gestational hypertension, pre-eclampsia, gestational diabetes, or mode of delivery. Only limited evidence was available incidentally from other studies. For example, Sommerhauser et al. reported some pregnancy complications and delivery modes among CCS using ART, but these outcomes were described only within the ART group and lacked comparison to spontaneous conceptions or population controls [16]. Overall, the paucity of dedicated studies in this area represents an important gap in the literature, particularly given the elevated baseline risks of obstetric complications observed both in cancer survivors and among those conceiving through ART [34]. Further research is needed to clarify these risks and to support informed counselling on maternal health for CCS*.*

### Strengths

This scoping review provides a comprehensive synthesis of the current literature on the use of ART in CCS, with a focus on fertility, maternal, perinatal, and child health outcomes. To our knowledge, this is the first review to systematically map the breadth of available evidence across these intersecting domains in this high-risk population. The review adhered to the PRISMA-ScR guidelines and employed a rigorous and transparent methodology, including a comprehensive search strategy across multiple databases (MEDLINE, Embase, and CINAHL), the use of both keywords and MeSH terms, and predefined inclusion criteria aligned with survivorship definitions used in the field. Screening and data extraction were conducted independently by three trained reviewers, minimising the risk of selection and extraction bias. The inclusion of studies on both male and female survivors, as well as diverse ART modalities including IVF, ICSI, and OTC, adds to the breadth and relevance of the findings. By identifying key gaps in the literature, such as the lack of data on obstetric complications and long-term child health outcomes, this review also provides a valuable foundation for future research and evidence-based fertility counselling for CCS.

### Limitations

Some limitations related to the exclusion criteria should be noted. The study was restricted to individuals diagnosed with cancer before the age of 18 years, in alignment with standard definitions of childhood cancer survivorship to ensure consistency across the included populations [35]. While this criterion enhances methodological clarity and aligns with existing literature, it may have excluded relevant data on survivors diagnosed in late adolescence or early adulthood, particularly those with ambiguous survivorship classification. Although not explored in the current review, future research could extend these considerations to young adult cancer survivors, who may face similar long-term health outcomes. Similarly, individuals currently undergoing treatment or receiving palliative care were excluded to focus on post-treatment survivorship; however, this may overlook transitional experiences that could inform fertility and reproductive outcomes. The review also excluded studies that did not report on the use or engagement with ART or failed to explicitly address ART utilisation among CCS as the primary outcome. Although this ensured the review remained targeted, it may have omitted studies with relevant secondary findings or indirect insights into reproductive health for CCS more generally. Furthermore, studies that did not include birth, perinatal, or obstetric outcomes were excluded, which may limit the review’s ability to capture broader reproductive implications of ART in this population. Case reports and case studies were not included as they can introduce limited generalisability; however, their exclusion may have added to the omission of rare but informative clinical presentations. Lastly, while systematic and narrative reviews were retained for background synthesis, they were not eligible for inclusion in the final analysis. These exclusions, while methodically justified, may have collectively narrowed the scope of the review and potentially excluded relevant sources of evidence.

## Conclusion

The use of ART provides a critical option for addressing infertility among CCS, a population at elevated risk of treatment-related reproductive impairment. While current evidence indicates that ART success rates in CCS are comparable to those in the general population, survivors exposed to pelvic or cranial radiotherapy may experience a reduced likelihood of achieving a live birth. Additionally, increased risks of low birth weight, preterm delivery, and multiple gestations have been observed in ART-conceived offspring in CCS, although no increased risk of congenital anomalies has been reported.

Despite these insights, existing studies are limited by small sample sizes, heterogeneous methodologies, and a narrow focus on short-term outcomes, with few examining follow-ups beyond the perinatal period. The lack of data on long-term maternal and child health outcomes, particularly in under-represented populations, including ethnic minorities and socioeconomically disadvantaged survivors in low-resource settings, restricts the broader applicability of findings.

To address these gaps, future research should prioritise longitudinal, adequately powered studies with standardised outcome measures, inclusive of diverse populations. Integration of early, personalised fertility counselling into survivorship care pathways is essential to optimise reproductive planning and ensure equitable access to fertility preservation and ART services.

## Supplementary Information

Below is the link to the electronic supplementary material.

ESM 1 (DOCX 15.6 KB)

## Data Availability

No datasets were generated or analyzed during the current study.
