# Supplementary material for: Infertility, the outcomes of assisted reproductive technologies use in childhood cancer survivors: a scoping review
Source: J Cancer Surviv. 2026 Feb 16;20(Suppl 2):2337–49. doi: 10.1007/s11764-026-01988-5 (PMC13744543; doi:10.1007/s11764-026-01988-5)
Supplement: Supplementary file 1 [file 11764_2026_1988_MOESM1_ESM.docx]

## **Infertility and the outcomes of assisted reproductive technologies use in childhood cancer survivors: A scoping review**

*Journal of Cancer Survivorship*

Elizabeth Sorial¹*, Leah Roberts¹, Erin Kelty¹, Sarah V. Ward², Ying Ru Feng¹ and David B. Preen¹

¹ School of Population and Global Health, The University of Western Australia, 35 Stirling Hwy, Crawley WA 6009

² UWA Medical School, The University of Western Australia, 35 Stirling Hwy, Crawley WA 6009

*Corresponding author. School of Population and Global Health, The University of Western Australia, 35 Stirling Hwy, Crawley WA 6009. E-mail: Elizabeth.sorial@research.uwa.edu.au

## **Supplementary materials**

| **Concept** | **Search Terms and Synonyms (Full List)** |
| --- | --- |
| Population: Childhood cancer survivors | Childhood cancer survivors and paediatric cancer survivors, including terms such as "childhood cancer survivors," "paediatric cancer," "adolescent cancer," "early-onset cancer," and related synonyms. ("Childhood cancer survivors" OR ((cancer? adj2 (child* OR "early onset" OR adolescen*)) AND (survivor? OR treatment OR chemotherapy OR radiotherapy))) Cancer Survivors/ child* OR "early onset" OR adolescen. |
| Intervention/Exposure: Fertility and ART | Fertility and ART-related interventions, including "assisted reproductive technology," "in vitro fertilisation (IVF)," "intracytoplasmic sperm injection (ICSI)," "intrauterine insemination (IUI)," "frozen embryo transfer (FET)," "fertility preservation," "ovarian reserve," "ovarian tissue cryopreservation," "reproductive function," "infertility treatment." ("assisted reproductive technology" OR ((pregnancy OR birth OR obstetric) AND outcomes) OR ((preservation OR treatment) AND fertility) OR "in vitro fertilisation" OR IVF OR "intrauterine insemination" OR IUI OR "intracytoplasmic sperm injection" OR ICSI OR "frozen embryo transfer" OR FET OR "ovarian reserve" OR "reproductive function" Pregnancy Outcome/ OR Infertility/ OR Infertility, Female/ OR Infertility, Male/ OR Reproductive Techniques, Assisted/ OR Fertility. |
| Outcomes: Reproductive and perinatal | Reproductive and perinatal outcomes, including "pregnancy outcomes," "birth outcomes," "perinatal outcomes," "maternal health," "neonatal health," "congenital anomalies," and "child health." |
| MeSH/Database-specific terms (PubMed) | ("paediatrics"[All Fields] OR "pediatrics"[MeSH Terms] OR "pediatrics"[All Fields] OR "paediatric"[All Fields] OR "pediatric"[All Fields]) AND ("femal"[All Fields] OR "female"[MeSH Terms] OR "female"[All Fields] OR "females"[All Fields] OR "female s"[All Fields] OR "femals"[All Fields]) AND ("cancer s"[All Fields] OR "cancerated"[All Fields] OR "canceration"[All Fields] OR "cancerization"[All Fields] OR "cancerized"[All Fields] OR "cancerous"[All Fields] OR "neoplasms"[MeSH Terms] OR "neoplasms"[All Fields] OR "cancer"[All Fields] OR "cancers"[All Fields] OR ("cysts"[MeSH Terms] OR "cysts"[All Fields] OR "cyst"[All Fields] OR "neurofibroma"[MeSH Terms] OR "neurofibroma"[All Fields] OR "neurofibromas"[All Fields] OR "tumor s"[All Fields] OR "tumoral"[All Fields] OR "tumorous"[All Fields] OR "tumour"[All Fields] OR "neoplasms"[MeSH Terms] OR "neoplasms"[All Fields] OR "tumor"[All Fields] OR "tumour s"[All Fields] OR "tumoural"[All Fields] OR "tumourous"[All Fields] OR "tumours"[All Fields] OR "tumors"[All Fields]) OR "neoplasms"[All Fields]) AND ("chemotherapy s"[All Fields] OR "drug therapy"[MeSH Terms] OR ("drug"[All Fields] AND "therapy"[All Fields]) OR "drug therapy"[All Fields] OR "chemotherapies"[All Fields] OR "drug therapy"[MeSH Subheading] OR "chemotherapy"[All Fields] OR ("radiotherapy"[MeSH Terms] OR "radiotherapy"[All Fields] OR "radiotherapies"[All Fields] OR "radiotherapy"[MeSH Subheading] OR "radiotherapy s"[All Fields])) AND ("cryopreservability"[All Fields] OR "cryopreservable"[All Fields] OR "cryopreservant"[All Fields] OR "cryopreservants"[All Fields] OR "cryopreservated"[All Fields] OR "cryopreservation"[MeSH Terms] OR "cryopreservation"[All Fields] OR "cryopreserved"[All Fields] OR "cryopreservations"[All Fields] OR "cryopreservative"[All Fields] OR "cryopreservatives"[All Fields] OR "cryopreserve"[All Fields] OR "cryopreserving"[All Fields] OR ("fertiles"[All Fields] OR "fertility"[MeSH Terms] OR "fertility"[All Fields] OR "fertile"[All Fields] OR "fertilities"[All Fields]) OR "fertility preservation"[All Fields]). |

## Supplementary 1 Search terms
